# Research on the Influence Mechanism of Enterprises’ Participation in School Enterprise Cooperation Based on the Analysis Framework of Theory of Planned Behavior

**DOI:** 10.3389/fpsyg.2022.860045

**Published:** 2022-03-30

**Authors:** Yuanbao Zhang, Junbin Wang, Xiangdong Shen, Jinyu Song

**Affiliations:** ^1^School of Education Science, Nanjing Normal University, Nanjing, China; ^2^Business School, Changshu Institute of Technology, Changshu, China; ^3^School of Electronic and Information Engineering, Changshu Institute of Technology, Changshu, China

**Keywords:** enterprise, school-enterprise cooperation, influence mechanism, Theory of Planned Behavior, behavior intention

## Abstract

School enterprise cooperation, as the basic school running form of applied undergraduate education, is an important way to cultivate applied talents. However, at present, the lack of motivation for enterprises to participate in school enterprise cooperation and the resulting problem of “school hot and enterprise cold” seriously limit the talent training quality in China’s application-oriented universities. There is an urgent need to explore the influencing factors and mechanisms of enterprises’ participation in school enterprise cooperation to improve the training quality of applied talents. Taking Ajzen (2002) Theory of Planned Behavior as the theoretical framework, this study constructs the influencing factor model of enterprise participation in school enterprise cooperation from four aspects, namely, behavior attitude, subjective norms, perceived behavior control, and behavior intention. In this study, participants (*N* = 250) completed a questionnaire assessing their participation in school enterprise cooperation, which was analyzed by a structural equation model. The results show that the behavior attitude and perceived behavior control of enterprises have a significant positive impact on their intention to participate in school enterprise cooperation and then have a significant positive impact on the school enterprise cooperation behavior of enterprises. The behavior intention and perceived behavior control of enterprises have a significant positive impact on their participation in school enterprise cooperation. The policy environment has a significant regulatory effect on the relationship between the intention and behavior of enterprises’ participation in school enterprise cooperation. Therefore, from the perspective of enhancing the intention of enterprises to cooperate, colleges and universities should establish the awareness of win-win cooperation and meet the interest demands of enterprises in school enterprise cooperation in order to improve the behavior attitude and intention of enterprises. From the perspective of the formation conditions of school enterprise cooperation, with the help of industry associations, an information service platform for school enterprise cooperation should be built in order to eliminate the information islands between enterprises and universities. From the perspective of the needs of school enterprise cooperation environment, government departments should strengthen the policy support for school enterprise cooperation in order to eliminate the worries of enterprises’ participation in school enterprise cooperation.

## Introduction

School enterprise cooperation is a talent training mode combining educational activities with social production practice, which uses schools, enterprises, social services, and other places for combined education, organically connecting students’ theoretical knowledge learning with production practice in order to realize the matching of talent training with social needs. The previous studies show that colleges and universities are no longer the only subjects of knowledge production with the transformation from knowledge production mode I to knowledge production mode II. School enterprise cooperation has become an important form of knowledge production (Wu et al., 2021). According to the Chinese government’s “opinions on promoting the high-quality development of modern vocational education,” school enterprise cooperation is an important way to promote the transformation of applied colleges and universities, and it is also a common way for the world’s higher education to pursue high-quality development. It plays a positive role in accelerating the cross-border integration of resources, stimulating students’ interest in learning, optimizing the professional structure of colleges and universities, promoting the transformation of scientific and technological achievements, and encouraging the coordinated development of education and industry. For example, the “dual system” model in Germany, the “cooperative education” model in the United States, the “official Industry School” model in Japan, and the “teaching factory” model in Singapore are known as successful models of school enterprise cooperative talent training, which have had a far-reaching impact on the world’s higher education.

Although school enterprise cooperation has been widely recognized by the society to promote the coordinated development of education and economy, and some countries have even raised school enterprise cooperation to the national strategic level, there are still many problems in reality, which are highlighted as follows: insufficient effective demand for enterprises to participate in school enterprise cooperation, lack of cooperation power, low level of cooperation problems, and single cooperation content (Liang et al., 2021). It is urgent to take the intention and behavior of enterprises to participate in school enterprise cooperation as the starting point to analyze the main factors affecting enterprises’ participation in school enterprise cooperation and its mechanism.

Enterprises’ participation in school enterprise cooperation is essentially a dynamic decision-making process that comprehensively considers social, political, economic, cost, and income factors. From the perspective of economics, as an “economic man,” an enterprise is essentially a kind of profit-making social organization, and its decision-making is always aimed at maximizing interests. Before enterprises decide to participate in school enterprise cooperation, they need to conduct a comprehensive evaluation from their own needs, cost investment, cooperation risk, short-term income, long-term income, and market factors (Gambin et al., 2010; Wang and Zhu, 2020). From the perspective of sociology, as a “social person,” an enterprise is an important part of the national economy, and any behavior of enterprise will be constrained by social responsibility and ethics. Before making the decision to participate in school enterprise cooperation, it will also conduct a comprehensive evaluation from the aspects of public expectation, social responsibility, social public opinion, peer evaluation, value recognition, social norms, and moral constraints (He and Su, 2021). In addition, enterprises’ participation in school enterprise cooperation is also affected by their own ability, economic environment, market information, resource conditions, and policy environment (Powers and Campbell, 2011). Therefore, it is difficult to explain the decision-making behavior of enterprises’ participation in school enterprise cooperation from any single perspective. Therefore, we used Ajzen’s Theory of Planned Behavior (TPB) model as our theoretical analysis framework (Ajzen, 1991).

Theory of Planned Behavior (TPB) is an important theoretical basis for explaining and predicting human behavior in the field of social psychology. It is widely used in the field of social psychology to explain and predict various human behaviors (Botetzagias et al., 2015; Echegaray and Hansstein, 2017; Allen and Marquart-Pyatt, 2018; Taufique and Vaithianathan, 2018). This study discusses several related issues affecting enterprises’ participation in school enterprise cooperation decision-making behavior, i.e., the impact of enterprises’ behavior attitude, subjective norms, perceived behavior control, and behavior intention on enterprises’ participation in school enterprise cooperation behavior. Ran (2021) believed that TPB model can predict the behavior of enterprises’ participation in school enterprise cooperation because enterprises often make a rough assessment according to their own needs, costs, benefits, risks, external pressure, and other factors when making any decision and then make a reasonable judgment on the behavior decision. At the same time, Sutton (1998) also pointed out that the degree of explanation of behavior intention by behavior attitude, subjective norms, and perceived behavior control (40–50%) is significantly higher than that of individual actual behavior (19–38%). In other words, there may be situational adjustment variables between behavior intention and actual behavior. However, the existing research lacks attention to this aspect. In this sense, the previous literature using TPB model to investigate the influencing factors of school enterprise cooperation is limited in explaining enterprise participation behavior.

In general, this study aims to integrate the perspective of TPB model and combine the operation law of school enterprise cooperation to explore the antecedents and regulation mechanism of enterprises’ participation in school enterprise cooperation. Specifically, this study mainly attempts to answer the following questions: (1) Will behavior attitude, subjective norms, and perceived behavior control affect enterprises’ intention to participate in school enterprise cooperation? (2) Will behavior intention and perceived behavior control affect enterprises’ participation in school enterprise cooperation? (3) Does the policy environment regulate the intention and behavior of enterprises’ participation in school enterprise cooperation? The answers to these questions help to reasonably explain the formation mechanism of enterprises’ participation in school enterprise cooperation in order to promote colleges and universities to continuously deepen school enterprise cooperation. Hence, this study uses the Theory of Planned Behavior as the analysis framework, accepts Chinese Suzhou enterprises as the investigation object, and proposes theoretical hypothesis based on previous studies. With the help of SPPS and AMOS research tools, it systematically analyzes and explains the formation mechanism of enterprises’ participation in school enterprise cooperation and proposes the corresponding countermeasures and suggestions.

## Theoretical Model and Hypothesis Development

### Theoretical Model

**FIGURE 1:**
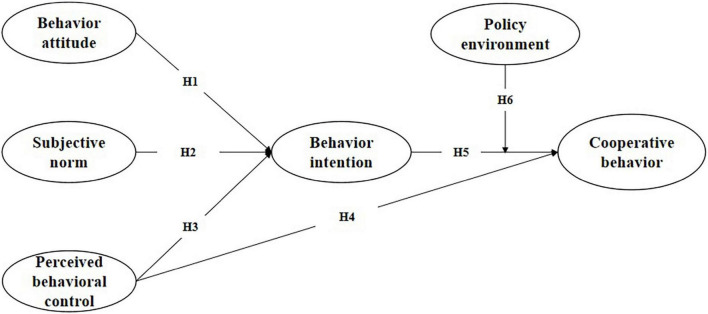
Research model.

Theory of Planned Behavior (TPB) is an important theory used to explain and predict behavioral decision-making in the field of social psychology. Since the formal proposal of Ajzen (1985), this theory has been widely used in human behavior research, and many studies have proved that this theory has a strong role in explaining and predicting the behavior intention and actual behavior of actors (Wang and Zhang, 2017). The behavior decision-making of the behavior subject is usually rational. By systematically collecting, analyzing, and using relevant information, we can fully consider whether to perform a specific behavior and the impact after the behavior occurs (Ajzen, 1991). Organizational behavior is determined by the behavior intention of core members, and behavior intention is affected by behavior attitude, subjective norms, and perceived behavior control (Ajzen, 2002). In general, the behavior of enterprises’ participation in school enterprise cooperation will be affected by many factors, e.g., enterprises’ cognition of cooperation objects, preferences of core management, public opinion pressure, their own resource conditions, corporate social reputation, government behavior, and expected risks and benefits. From the perspective of colleges and universities, to attract more high-quality enterprises to participate in school enterprise cooperation, we must clearly understand what factors influence the behavior and decision-making of enterprises. According to the actual situation of school enterprise cooperation and existing research results, the behavior attitude, behavior norms, perceived behavior control, and external policy environment of the core management of enterprises will have an impact on the decision-making of school enterprise cooperation. Therefore, a model was proposed (Figure 1).

### Hypothesis Development

#### Behavior Attitude and Behavior Intention

Behavior attitude refers to the degree to which an individual likes or dislikes the implementation of a specific behavior (Ajzen, 1991). When the behavior subject decides whether he or she is willing to perform a specific behavior, he or she will subjectively evaluate the results of the behavior (Cheng et al., 2006). When the behavior subject takes a positive attitude toward the consequences of this specific behavior, he or she will be willing to spend more time and energy to perform this specific behavior (Mahagaonkar, 2010). Therefore, how to improve the behavior attitude of enterprises is a problem that enterprises must pay attention to when they participate in school enterprise cooperation. There have been a lot of studies on the antecedents of trust. Franco and Haase (2015) found that there is a significant correlation between the behavior attitude of enterprises and the intention of enterprises’ participation in school enterprise cooperation. In terms of intention to participate in school enterprise cooperation, enterprises will first make a subjective assessment according to their own needs, benefits, costs, risks, and other factors. When enterprises hold a positive attitude toward this, their intention to participate will be strong. Therefore, this study proposes the following hypothesis:

H1. Behavior attitude has a positive impact on enterprises’ intention to participate in school enterprise cooperation.

#### Subjective Norms and Behavior Intention

Subjective norms are the external pressure that individuals feel before performing a specific behavior, especially various social relations in the social structure (Ajzen, 1991). Due to the existence of norms, while pursuing the maximization of interests, enterprises also need to accept the constraints of ethics, ideals and beliefs, social responsibility, social expectations, and social public opinion. Carrasco (2007) found that carrying out cooperative education and research with colleges and universities is an important social responsibility entrusted by the society to enterprises, and this sense of responsibility will affect the intention of enterprises’ participation in school enterprise cooperation (Venkatesh and Davis, 2000). In addition, the exemplary norms of reference groups will also have an impact on the formation of subjective standards of behavior subjects, especially small, and medium-sized enterprises. When enterprises with similar development experience or leading enterprises in their peers achieve certain results through school enterprise cooperation, they are willing to learn their growth path and regulate their own behavior according to the standards of the reference group (Fisher, 2004). In other words, the greater the subjective norms of enterprises, the stronger the intention to participate in school enterprise cooperation. Therefore, this study proposes the following hypothesis:

H2. Subjective norms have a positive impact on the behavior intention of enterprises’ participation in school enterprise cooperation.

#### Perceived Behavior Control and Behavior Intention

Perceived behavior control reflects an individual’s unconscious complete control behavior. It is the degree of difficulty that an individual perceives and executes a specific behavior. The stronger the behavior subject’s perceived behavior control ability, the stronger its intention to perform a specific behavior (Kraft et al., 2005). In general, before deciding to cooperate with colleges and universities, enterprises will always pre-evaluate various external obstacles, including their own resources, market competition environment, information occupancy, opportunities, and expected resistance factors. When an enterprise feels that it can control these external influencing factors, it is more likely to perform this behavior (Zhang et al., 2021). In other words, even if the behavior attitude and subjective norms of enterprises’ participation in school enterprise cooperation are positive, enterprises will not have a strong intention to participate if they do not have objective implementation conditions. Therefore, this study proposes the following hypothesis:

H3. Perceived behavior control has a positive impact on enterprises’ behavior intention to participate in school enterprise cooperation

#### Behavior Intention and Cooperative Behavior

Behavior intention refers to the subjective probability of an individual implementing a specific behavior or the degree of effort he or she is willing to invest (Elliott et al., 2007). Krueger et al. (2000) found that there is a significant positive correlation between individual behavior intention and behavior. Individual behavior intention is the direct antecedent variable of their behavior, especially for those uncommon, and difficult to observe behaviors, behavior intention has been proved to be the most effective predictor of behavior. Chen (2017) found that enterprises with strong intention are more willing to actively fulfill their obligations in the process of school enterprise cooperation and publicize the positive role of school enterprise cooperation. In other words, enterprises’ participation in school enterprise cooperation is essentially a rational and planned behavior. The stronger their behavior intention, the greater the possibility of participating in school enterprise cooperation. Therefore, this study proposes the following hypothesis:

H4. Behavior intention has a positive impact on enterprises’ participation in school enterprise cooperation.

#### Perceived Behavior Control and Cooperative Behavior

Perceived behavior control cannot only directly affect behavior intention but also directly affect individual behavior. When the behavior to be predicted is not completely under conscious control or perceived behavior control reflects the actual control to a certain degree, perceived behavior control can have a direct impact on individual behavior without behavior intention (Ajzen and Sexton, 1999). Ran (2021) found that there is a significant correlation between perceived behavior control and enterprise participation in school enterprise cooperation. When enterprises have more resources, more opportunities, and fewer expected obstacles to participate in school enterprise cooperation, the more enterprises can stimulate their participation behavior. Therefore, this study proposes the following hypothesis:

H5. Perceived behavior control has a positive impact on enterprises’ participation in school enterprise cooperation.

#### Policy Environment, Behavior Intention, and Cooperative Behavior

There is not a simple linear relationship between behavior intention and behavior, but there are situational adjustment variables between them. Armitage and Conner (2001), by meta-analysis of 185 studies in literature on planned behavior theory, found that behavior intention can only explain 16–37% of behavioral variance. In the school enterprise cooperation system, the government, universities, and enterprises are essentially a “three spiral” relationship, and the policy environment will also affect the participation behavior of enterprises (Etzkowitz, 2016). This is because, on the one hand, public policies may enhance the social reputation of enterprises through incentives in order to promote enterprises to transform their behavior intention into practical action (Muehlemann et al., 2007); on the other hand, it can also effectively reduce enterprise speculation and promote the achievement of school enterprise cooperation by regulating the leading role (Ryan et al., 2007). In addition, Huergo and Moreno (2017) found that the government’s policy support, financial subsidies, and tax incentives have a significant regulatory effect on enterprises’ intention and behavior to participate in university cooperative innovation. Especially in China, with the school enterprise cooperation mode of “government leading, University subject and enterprise participation,” the impact of policy environment on enterprise participation is often more obvious. Therefore, this study proposes the following hypothesis:

H6. Enterprises’ behavior intention and participation in school enterprise cooperation are positively regulated by the policy environment.

## Research Methodology

### Instrument

To ensure the scientificity and effectiveness of the questionnaire, all items used for measurement are adapted from the existing literature and modified appropriately according to the research purpose and the actual situation of the research object. The project used to measure enterprises’ behavior attitude and subjective behavior norms was adapted from the study by Gu et al. (2018). The project for measuring perceived behavior control of enterprises was adapted from the study by Huang and Zheng (2015). The project for measuring behavior intention of enterprises was adapted from the study by Feng and Zhang (2020). The project for measuring enterprises’ cooperative behavior was adapted from the research by Ran (2021). Finally, three items used to measure situational factors were adapted from the study by Li et al. (2014). The final questionnaire is presented in Appendix Table 1. All items were measured using the 5-point Likert scale, ranging from 1 (very disagree) to 5 (very agree).

### Data Collection

The survey object of this study is Suzhou enterprises in China. The reasons for choosing Suzhou enterprises are as follows: Suzhou is one of the important central cities in China’s Yangtze River Delta, one of the cities with the most complete industrial system in China, a high-tech industrial base in China with more than 160,000 industrial enterprises. A questionnaire for this study was published on sojump, China’s largest online survey platform. Core members with certain decision-making rights, mainly including the president, general manager, deputy general manager, personnel manager, chief financial officer, marketing director, and technical backbone, are invited to participate in the filling. The data of this study were collected in December 2021. In the survey sample, 78.40% of enterprises have been registered for less than 20 years, and 82.00% of enterprises have a scale of less than 1,000 people. Similar to enterprise ownership, private enterprises account for 42.40%, followed by sole proprietorship enterprises and state-owned enterprises, accounting for 17.60 and 16.00%, respectively. The types of enterprises mainly include the manufacturing industry, biological pharmaceutical industry, automobile service industry, and commerce and construction industry. In general, the survey sample comprehensively considers the years of enterprise registration, the size of employees, the nature of ownership, and the type of industry, which is well representative (Table 1).

**TABLE 1:** Demographics of the survey respondents (*N* = 250).

Demographic	Category	Frequency	%
Registration time	≤5	74	29.6
	6–10	32	12.8
	10–19	90	36.0
	≥20	54	21.6
Staff size	≤99	130	52.0
	100–199	34	13.6
	200–499	24	9.6
	500–999	17	6.8
	≥1,000	45	18.0
Ownership form	State-owned enterprise	40	16.0
	Private enterprise	106	42.4
	Sole proprietorship	44	17.6
	Hong Kong, Macao, and Taiwan investment enterprises	15	6.0
	Foreign enterprise	24	9.6
	Other	21	8.4
Industry type	Manufacturing	108	43.2
	Construction	16	6.4
	IT industry	11	4.4
	Commerce	17	6.8
	Automobile industry	26	10.4
	Pharmaceuticals	55	22.0
	Other	17	6.8

## Data Analysis and Results

### Reliability and Validity

Construct reliability and validity were further examined through CFA. As shown in Table 2, the Cronbach’s α, and composite reliability (CR) values for each construct ranged from 0.910 to 0.973, both of which were above the suggested threshold of 0.7 (Straub et al., 2004) and exhibited a satisfactory level of reliability. For construct validity, both convergent validity and discriminant validity were examined. Convergent validity was confirmed by examining both the average variance extracted (AVE) and indicator loadings. As shown in Table 2, all AVE values were higher than the recommended level of 0.5 (Fornell and Larcker, 1981). The standard loadings of all items were above the desired threshold of 0.7 and statistically significant at 0.001. This indicated good convergent validity (Chin et al., 1997).

**TABLE 2:** Results of confirmatory factor analysis.

Construct	Indicator	Standard loading^a^	Cronbach’s α	CR	AVE
Behavior attitude	BEA1	0.894	0.962	0.966	0.903
	BEA 2	0.984			
	BEA 3	0.971			
Perceived behavioral control	PBC1	0.894	0.960	0.960	0.858
	PBC2	0.923			
	PBC3	0.951			
	PBC4	0.936			
Subjective norm	SUN1	0.853	0.941	0.945	0.853
	SUN2	0.973			
	SUN3	0.940			
Behavior intention	BEI1	0.939	0.972	0.973	0.923
	BEI2	0.970			
	BEI3	0.972			
Cooperative behavior	COB1	0.967	0.942	0.948	0.859
	COB2	0.926			
	COB3	0.885			
Policy environment	POE1	0.877	0.910	0.918	0.790
	POE2	0.935			
	POE3	0.852			

*χ^2^ = 2.290, CFI = 0.981, TLI = 0.974, GFI = 0.910, NFI = 0.966, RMSEA = 0.072.*

*^a^All standard loadings were significant at p < 0.001.*

Discriminant validity was evaluated by comparing the square root of AVE and the correlation value. The discriminant validity was assessed by comparing the square root of AVE for each construct with the correlations between that construct and other constructs (Fornell and Larcker, 1981). As shown in Table 3, the square roots of the AVEs (i.e., diagonal elements) were larger than the inter-construct correlations depicted in the off-diagonal entries, thus suggesting the discriminant validity that was adequate.

**TABLE 3:** Results of discriminant validity testing.

	Mean	*S.D.*	BEA	PBC	SUN	BEI	COB	POE
BEA	4.587	0.786	***0.950***					
PBC	4.269	1.010	0.647	***0.926***				
SUN	4.384	0.916	0.787	0.657	***0.924***			
BEI	4.467	0.883	0.725	0.818	0.658	***0.961***		
COB	4.195	1.160	0.548	0.738	0.492	0.726	***0.927***	
POE	4.312	1.025	0.617	0.607	0.624	0.727	0.780	**0.889**

*BEA, Behavior attitude; PBC, Perceived behavioral control; SUN, subjective norm; BEI, behavior intention; COB, Cooperative behavior; POE, Policy environment. Diagonal bold italics entries are square root of AVE; all others are correlations coefficients. Bold values indicate the square root of AVE.*

As the data were self-reported from a single source, we made a statistical analysis of the data to assess common method bias. First, we further assessed the method factor according to the steps suggested by Liang et al. (2007). The results demonstrated that the loadings of the principal variables were all significant at the *p* < 0.001 level, whereas none of the common method factor loadings was significant. These results further indicated that common method bias was unlikely to be a concern in this study.

Second, we conducted a multicollinearity test to examine the correlations between the independent variables. A variance inflation factor (VIF) value above 10 indicates a multicollinearity problem. As shown in Table 4, the VIF values for the variables in this study were all below 10, indicating the absence of multicollinearity.

**TABLE 4:** Results of multicollinearity analysis.

	Unstandardized coefficient		Standardized coefficient			Multicollinearity statistics	
Model	B	Standard error	β	*T*	Significance	Tolerance	VIF
1(con.)^a^	–0.374	0.245		–1.524	0.129		
BEA	0.058	0.930	0.040	.624	0.533	0.304	3.290
PBC	0.420	0.070	0.364	5.967	0.000	0.337	2.968
SUN	–0.281	0.081	–0.221	–3.476	0.001	0.311	3.220
BEI	0.290	0.090	0.226	3.222	0.001	0.256	3.909
POE	0.567	0.060	0.487	9.509	0.000	0.477	2.096

*^a^Dependent variable: Cooperative behavior; BEA, Behavior attitude; PBC, Perceived behavioral control; SUN, subjective norm; BEI, behavior intention.*

### Hypothesis Testing

**FIGURE 2:**
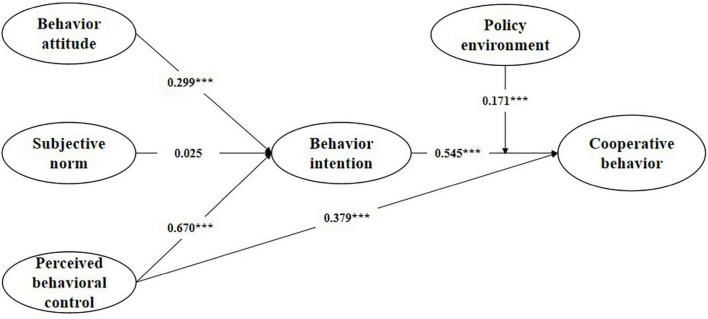
The results of the research model. *p < 0.05, **p < 0.01, ***p < 0.001.

Figure 2 indicates that behavior attitude and perceived behavior control have a positive impact on behavior intention, which supports hypotheses H1 and H3. Behavior intention and perceived behavior control have a positive impact on participation behavior, thus supporting hypotheses H4 and H5. Subjective norms have no significant impact on behavior intention, so they reject hypothesis H2. Corporate behavior intention and participation in school enterprise cooperation are positively regulated by the policy environment (β = 0.171, *p* < 0.001) (Table 5), thus supporting H6. Among the two antecedents of behavior intention, perceived behavior control has a greater impact on behavior intention (β = 0.670, *p* < 0.001), followed by behavior attitude (β = 0.299, *p* < 0.001). Among the two antecedents of participation behavior, behavior intention has a greater impact on participation behavior (β = 0.545, *p* < 0.001), followed by perceived behavior control (β = 0.379, *p* < 0.001) (Table 6).

**TABLE 5:** Adjustment variable analysis.

Adjustment variable path	Estimate	S.E.	C.R.	*P*
Behavior intention →Cooperative behavior	–0.020	0.174	–0.113	0.910
Policy environment →Cooperative behavior	–0.152	0.193	–0.788	0.431
Behavior intention* Policy environment →Cooperative behavior	0.171	0.047	3.673	***

*p<0.05; **p<0.01; ***p<0.001.

**TABLE 6:** Hypotheses test.

Hypothesis path	Path coefficient	*S.E.*	*t*-value	*p*-value	Results
H1: Behavior attitude → Behavior intention	0.299	0.058	5.131	***	Supported
H2: Subjective norm → Behavior intention	0.025	0.050	0.505	0.614	Unsupported
H3: Perceived behavioral control → Behavior intention	0.670	0.046	14.445	***	Supported
H4: Behavior intention → Cooperative behavior	0.545	0.102	5.362	***	Supported
H5: Perceived behavioral control → Cooperative behavior	0.379	0.099	3.820	***	Supported

*p<0.05; **p<0.01; ***p<0.001.

Next, we examined trust in platform and satisfaction mediation effects using the bootstrapping approach provided by Preacher and Hayes (2008). The use and test of mediating effect is the main trend in management studies. Table 7 shows that behavior intention plays a complete intermediary role between behavior attitude and participation behavior, and behavior intention plays a partial intermediary role between subjective perception norms and participation behavior, with a 95% bootstrap confidence interval, excluding 0. This finding suggests that behavior intention mediates the effect of behavior attitude, perceived behavioral control on the enterprise cooperative behavior.

**TABLE 7:** Results of mediating effect analysis.

IV	M	DV	IV→su	IV→sul	MV→su	Indirect effect	Cls	Mediation
BEA	BEI	COB	0.721***	0.050	0.694***	0.497*** (0.052)	[0.349, 0.681]	Yes
PBC			0.818***	0.440***	0.367***	0.300* (0.094)	[0.063, 0.593]	Yes

95% Bootstrap CIs for the indirect effect. IV, independent variable; M, mediator variable; DV, dependent variable; BEA, Behavior attitude; PBC, Perceived behavioral control; BEI, behavior intention; COB, Cooperative behavior. IV → DV is significant (M not included in the model); IV → M is significant; M → DV is significant (or the meaningful reduction in effect) of the relationships between the initial IV and DV in the presence of mediator. Significance at *p < 0.05, **p < 0.01, and ***p < 0.001; SEs in brackets.

## Discussion and Implications

### Discussion of Findings

This study yielded interesting findings. As the results indicate that behavior attitude, subjective norms, and perceived behavior control have different degrees of impact on enterprises’ intention to participate in school enterprise cooperation and actual participation behavior. First, behavior attitude and perceived behavior control have a significant positive impact on enterprises’ intention to participate in school enterprise cooperation. Behavior attitude have a stronger effect on behavior intention (β = 0.299, *p* < 0.001), and perceived behavioral control have stronger effects on behavior intention (β = 0.379, *p* < 0.001). Moreover, subjective norms do not significantly affect the enterprises’ behavior intention to participate in school enterprise cooperation. The possible reason is that enterprises, as profit-making social organizations, think more about their own interests and less about the subjective normative pressure from similar enterprises, industry associations, and news media. This also confirms Li’s (2021) view that expected income is the primary driving force to stimulate enterprises to participate in school enterprise cooperation. In particular, small and medium-sized enterprises will further consider social norms only when their reasonable demands are met. In addition, the findings are consistent with those of the previous studies (Ran, 2021), indicating that the enterprises’ behavior intention to participate in school enterprise cooperation is mainly affected by behavior attitude and perceived behavior control.

Second, behavior intention and perceived behavior control have a significant positive impact on the actual behavior of enterprises’ participation in school enterprise cooperation. Regarding the effects of behavior intention and perceived behavioral control on cooperative behavior, our results indicate that behavior intention and perceived behavioral control can predict cooperative behavior and that behavior intention (β = 0.545, *p* < 0.001) plays a greater role in determining cooperative behavior than do perceive behavioral control (β = 0.379, *p* < 0.01). This suggests that behavior intention is the leading factor affecting enterprises’ participation in school enterprise cooperation. Our findings extend those of previous studies (Li et al., 2014), suggesting that behavior intention and perceived behavior control have the same effect on enterprises’ participation in school enterprise cooperation. Finally, this study confirms that the policy environment has a positive regulatory effect on the enterprises’ behavior intention to participate in school enterprise cooperation and the actual participation behavior. The interaction between policy environment and behavior intention has a significant positive impact on the actual behavior of enterprises’ participation in school enterprise cooperation (β = 0.171, *p* < 0.01). The hypothesis is verified to be in line with the literature, highlighting regulating effect of policy environment on behavior intention and cooperative behavior (Jing, 2019; He and Wu, 2020).

### Theoretical Contribution

This study helps to better explain the mechanism of enterprises’ participation in school enterprise cooperation through TPB model. Using the TPB model, many studies have explored the influences of subjective cognition of behavior subjects on inter-organizational cooperative behavior (Li et al., 2014). These studies show that the behavior attitude, subjective norms, and perceived behavior control of behavior subjects have a positive impact on the intention and behavior of school enterprise cooperation. However, most of these studies focus on the cooperative behavior of enterprises and its influencing factors from the perspective of “rational people” (Ran, 2017; Xiao and Chen, 2019), lack of exploration on the impact of situational factors on organizational behavior. Different from previous research conclusions, in addition to individual subjective cognitive factors, the policy environment also affects the decision-making behavior of enterprises to a great extent.

Based on the TPB model, four main factors that affect enterprises’ participation in school enterprise cooperation are determined, namely behavior attitude, perceived behavior control, behavior intention, and policy environment. Behavior attitude has a significant positive impact on enterprises’ participation in school enterprise cooperation through behavior intention. Perceived behavior control not only has a significant positive impact on enterprise behavior directly but also has a significant positive impact on enterprise behavior indirectly through behavior intention. The policy environment has a significant positive regulatory effect on the intention and behavior of enterprises to participate in school enterprise cooperation. This study not only supplements the academic literature on the generation mechanism of enterprises’ participation in school enterprise cooperation but also provides a new perspective for colleges and universities to evaluate the potential cooperation behavior of enterprises.

### Managerial Implications

This study offers useful managerial implications from two aspects. For the first time, with the transformation of knowledge production mode, school enterprise cooperation has increasingly become an important way for colleges and universities to implement applied talents’ cultivation. How to attract more high-quality enterprises to participate in school enterprise cooperation has become the focus of higher education in the world. This study found that enterprises’ behavior attitude, perceived behavior control, behavior intention, and policy environment have a significant positive impact on enterprises’ participation in school enterprise cooperation and then revealed the internal mechanism of school enterprise cooperation. For university managers, on the one hand, they should establish the cooperation concept of complementary advantages, resource sharing and mutual benefit and win-win, meet the reasonable interest demands of enterprises in school enterprise cooperation in order to improve the behavior attitude and behavior intention of enterprises, and then promote the achievement of marching cooperation. On the other hand, we should build an information service platform for school enterprise cooperation with the help of industry associations, unblock information communication channels, and eliminate information islands between enterprises and universities.

Second, the relationship among enterprises, universities, and the government is essentially a “three spiral” relationship in school enterprise cooperation. With the rapid expansion of the scale of higher education and the continuous dilution of school running resources, the effect of the government in the process of school enterprise cooperation is becoming more and more obvious. This study found that the policy environment has a significant regulatory effect on the intention and behavior of enterprises to participate in school enterprise cooperation. Therefore, optimizing the policy environment of industry education integration is another key point to comprehensively deepen school enterprise cooperation. For government managers, on the one hand, they should strengthen the upper and lower connection of school enterprise cooperation policies to ensure the systematic nature and unity between upper and lower policies of school enterprise cooperation. On the other hand, they should strengthen the policy support between the education department and the industrial department, as well as the coordination between the policy implementation subjects in order to form the internal and external resultant force of the school enterprise cooperation policy.

### Limitations and Future Research

Based on the TPB model, this study constructs a decision-making behavior model of enterprises’ participation in school enterprise cooperation and tests the model through the questionnaire data of enterprise core management. The results show that the constructed model is highly competent in explaining the problem, but this research still has the following limitations: first, this article selects Suzhou enterprises in economically developed areas as representatives, but enterprises in other economically relatively backward areas may have certain differences; second, this study does not consider the impact of cost, income, and risk on enterprises’ participation in school enterprise cooperation decision-making. In future research, income, cost, risk, and economic factors can be used as regulatory variables to compare the participation of enterprises in different regions in school enterprise cooperation.

## Data Availability Statement

The original contributions presented in the study are included in the article/Supplementary Material, further inquiries can be directed to the corresponding author/s.

## Ethics Statement

The studies involving human participants were reviewed and approved by the School of Business, Changshu Institute of Technology. Written informed consent for participation was not required for this study in accordance with the national legislation and the institutional requirements.

## Author Contributions

YZ designed the study and drafted the initial manuscript. YZ and XS collected the data, performed statistical analysis, and drafted the initial manuscript. JW and JS contributed to the revised manuscript. All authors discussed the results and contributed to the final manuscript.

## Conflict of Interest

The authors declare that the research was conducted in the absence of any commercial or financial relationships that could be construed as a potential conflict of interest.

## Publisher’s Note

All claims expressed in this article are solely those of the authors and do not necessarily represent those of their affiliated organizations, or those of the publisher, the editors and the reviewers. Any product that may be evaluated in this article, or claim that may be made by its manufacturer, is not guaranteed or endorsed by the publisher.

## Appendix

**APPENDIX TABLE 1:** Questionnaire items.

Construct	Item	Source
Behavior attitude	BEA1: It is necessary for enterprises to participate in school-enterprise cooperation	
	BEA2: It is beneficial for enterprises to participate in school-enterprise cooperation	
	BEA3: It is wise for enterprises to participate in school-enterprise cooperation	
Subjective norm	SUN1: Peer enterprises actively cooperate with the university and enterprise	
	SUN2: Industry associations support enterprises to participate in cooperation	
	SUN3: Public opinion advocates enterprises to participate in cooperation	
Perceived behavioral control	PBC1: The enterprise already has the school-enterprise cooperation experience	
	PBC2: Enterprises know the relevant information about participating in cooperation	
	PBC3: Enterprises can quickly establish cooperative relationships with universities	
	PBC4: Enterprises are confident to achieve the goal of participating	
Behavior intention	BEI1: The enterprise wishes to take an active part in school-enterprise cooperation	
	BEI2: The enterprise is willing to undertake the responsibilities and tasks in cooperation	
	BEI3: Companies are willing to advertise the positive effects of cooperation	
Cooperative behavior	COB1: Companies have partnered with universities in the past	Ran, 2021
	COB2: Companies are now cooperating with universities	
	COB3: Enterprises will further cooperate with universities in the future	
Policy environment	POE1: The state encourages enterprises to participate in cooperation	Li et al., 2014
	POE2: The state introduced policies to support school-enterprise cooperation	
	POE3: Enterprises enjoy the replenishment benefits of government policies	
